# Modulation of benzylisoquinoline alkaloid biosynthesis by overexpression berberine bridge enzyme in *Macleaya cordata*

**DOI:** 10.1038/s41598-018-36211-8

**Published:** 2018-12-20

**Authors:** Peng Huang, Wei Liu, Min Xu, Ruolan Jiang, Liqiong Xia, Peng Wang, Han Li, Zhaoshan Tang, Qiyin Zheng, Jianguo Zeng

**Affiliations:** 1 Hunan Key Laboratory of Traditional Chinese Veterinary Medicine, Hunan Agricultural University, Changsha, Hunan 410128 China; 2 College of Horticulture and Landscape, Hunan Agricultural University, Changsha, Hunan 410128 China; 3 Center of Analytic Service, Hunan Agricultural University, Changsha, Hunan 410128 China; 4 School of pharmacy, Hunan University of Chinese Medicine, Changsha, 410208 China; 5 State Key Laboratory of Silkworm Genome Biology, Southwest University, Beibei, Chongqing, 400715 China; 6 Micolta Bioresource Inc., Changsha, 410016 China; 7 National and Local Union Engineering Research Center of Veterinary Herbal Medicine Resource and Initiative, Hunan Agricultural University, Changsha, Hunan 410128 China

## Abstract

*Macleaya cordata* produces a variety of benzylisoquinoline alkaloids (BIAs), such as sanguinarine, protopine, and berberine, which are potential anticancer drugs and natural growth promoters. The genes encoding the berberine bridge enzyme (BBE) were isolated from *M. cordata* and *Papaver somniferum*, and then the two genes were overexpressed in *M. cordata*. Through liquid chromatography with triple-quadrupole mass spectrometry analysis, it was determined that McBBE-OX caused higher levels of (*S*)-norcoclaurine, (*S*)-coclaurine, (*S*)-*N*-cis-methylcoclaurine, (*S*)-reticuline, (*S*)-tetrahydrocolumbamine, (*S*)-tetrahydroberberine, (*S*)-cheilanthifoline, and (*S*)-scoulerine than PsBBE-OX, empty vector or control treatments. qRT-PCR analysis demonstrated that the introduced genes in the transgenic lines were all highly expressed. However, the levels of sanguinarine (SAN) and chelerythrine (CHE) in all the transgenic lines were slightly lower than those in the wild-type lines, possibly because the overexpression of *McBBE* causes feedback-inhibition. This is the first report on the overexpression of potential key genes in *M. cordata*, and the findings are important for the design of metabolic engineering strategies that target BIAs biosynthesis.

## Introduction

**Figure 1:**
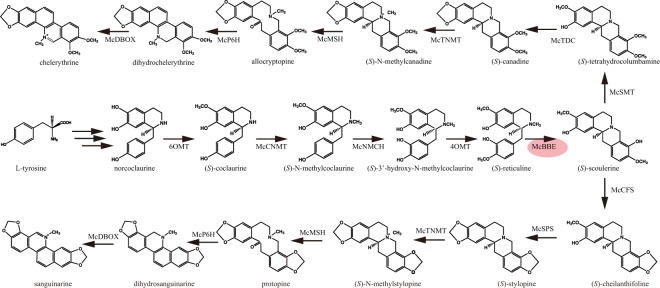
The metabolic pathway of sanguinarine and chelerythrine. Berberine bridge enzyme (BBE) was studied in this work. 6OMT, norcoclaurine 6-*O*-methyltransferase; CNMT, coclaurine-*N*-methyltransferase; NMCH, *N*-methylcoclaurine hydroxylase; 4OMT, 4′-*O*-methyltransferase; BBE, berberine bridge enzyme; CFS, cheilanthifoline synthase; SPS, stylopine synthase; TNMT, tetrahydroprotoberberine cis-*N*-methyltransferase; MSH, (*S*)-cis-*N*-methylstylopine 14-hydroxylase, P6H, protopine 6-hydroxylase; DBOX, dihydrobenzophenanthridine oxidase; TDC, (*S*)-canadine synthase; SMT, (*S*)-scoulerine 9-*O*- methyltransferase.

Benzylisoquinoline alkaloids (BIAs) are a large and structurally diverse group of natural products, most of which occur in the families Papaveraceae, Ranunculaceae, Lauraceae, Rutaceae and Menispermaceae^1^. Some BIAs have important clinical medicinal benefits; for example, morphine is a narcotic drug, berberine (BBR) has been used to treat bacterial diarrhoea^2^, and protopine (PRO) and sanguinarine (SAN) were potential anticancer drugs and have real potential as effective antischistosomal drugs^3–5^. More importantly, SAN has been widely used in livestock as an alternative to antibiotic growth promoters^6,7^. Recently, the market demand for BIAs has increased steadily every year. However, the use of BIAs is significantly restricted because of its low levels in plants. Fortunately, the synthetic pathways and enzymes of BIAs in the Papaveraceae family of plants have been elucidated in previous studies^8,9^. BIA biosynthesis begins with tyrosine; then, two tyrosine derivatives, dopamine and 4-hydroxyphenylacetaldehyde, are condensed to form (*S*)-norcoclaurine. Then, (*S*)-norcoclaurine is converted into (*S*)-reticuline through several steps. If we consider the BIA synthetic pathway to be a network, (*S*)-reticuline is the key branch-point intermediate of BIAs. This compound is located at a crucial crossroads site, and the *N*-methyl group of (*S*)-reticuline is converted into the methylene bridge moiety of (*S*)-scoulerine by the berberine bridge enzyme (BBE) (Fig. 1). In fact, (*S*)-scoulerine is the key intermediate in many BIA-producing plants, and BBE is the most critical rate-limiting enzyme in the entire BIA synthesis pathway^10^.

In recent years, many studies have engineered microbes and plants to produce BIAs^11–17^. Microbial-based production of BIAs requires the introduction of some heterologous plant genes and numerous genetic modifications to increase productivity. However, the advances in microbial-based methods have significantly shortened production time and although microbial methods are not affected by the environment, the production of BIAs still needs an external supply of precursors^15^. In a recent study, researchers combined different enzymes from various species and, through pathway and strain optimization, accomplished full opiate biosynthesis in yeast^16^. This work solved the problem of BIA production needing precursors, but the synthesis step was too long, and the final product concentration was hardly sufficient for industrial production. For this reason, many studies have used transgenic methods to increase production, especially overexpression of potential key enzymes. The most famous examples are the strategies used to increase artemisinin yield in *Artemisia annua*. Although semi-synthesis of artemisinin was successfully achieved in yeast^18^, many groups still enhance the yield of artemisinin by transgenic methods^19^. In addition, overexpression of synthesis genes has also been used to increase levels of ginsenoside, phytosterols, scopolamine and terpenoid indole alkaloids^20–22^. In addition, in a previous study, the content of BIAs in California poppy was increased through overexpression of the *P. somniferum BBE* gene (*PsBBE*), and this experiment indicated that BBE is a potential key enzyme to enhance the content of BIAs^12^.

*Macleaya cordata* is a perennial herb that belongs to the Papaveraceae family and is used for the commercial production of BIAs, including SAN, PRO, BBR; the herb contains many trace BIAs that have beneficial medicinal properties, such as (*S*)-reticuline and (*S*)-scoulerine. Recently, the whole genome of *M. cordata* has been de novo sequenced and validated for SAN biosynthesis^9^. In addition, a transformation and regeneration system to produce transgenic *M. cordata* has been constructed^23^. These genomics data and transgenic methods provide the opportunity for enhanced production of BIAs through plant metabolic engineering. In this study, we overexpressed *BBE* genes isolated from *M. cordata* and *P. somniferum* (*McBBE* and *PsBBE*) in *M. cordata*. Then, we investigated whether the overexpression of *McBBE* or *PsBBE* in *M. cordata* could affect the biosynthesis of BIAs. Finally, we investigated the metabolite profiles through ESI/QQQ MS analysis and detected the expression levels of 11 genes involved in the synthesis of SAN and CHE. To our knowledge, this is the first study using overexpression of BBE in transgenic *M. cordata* with increased alkaloid production.

## Results

### Establishment of Transgenic Plants with the *McBBE/PsBBE* Gene

**Figure 2:**
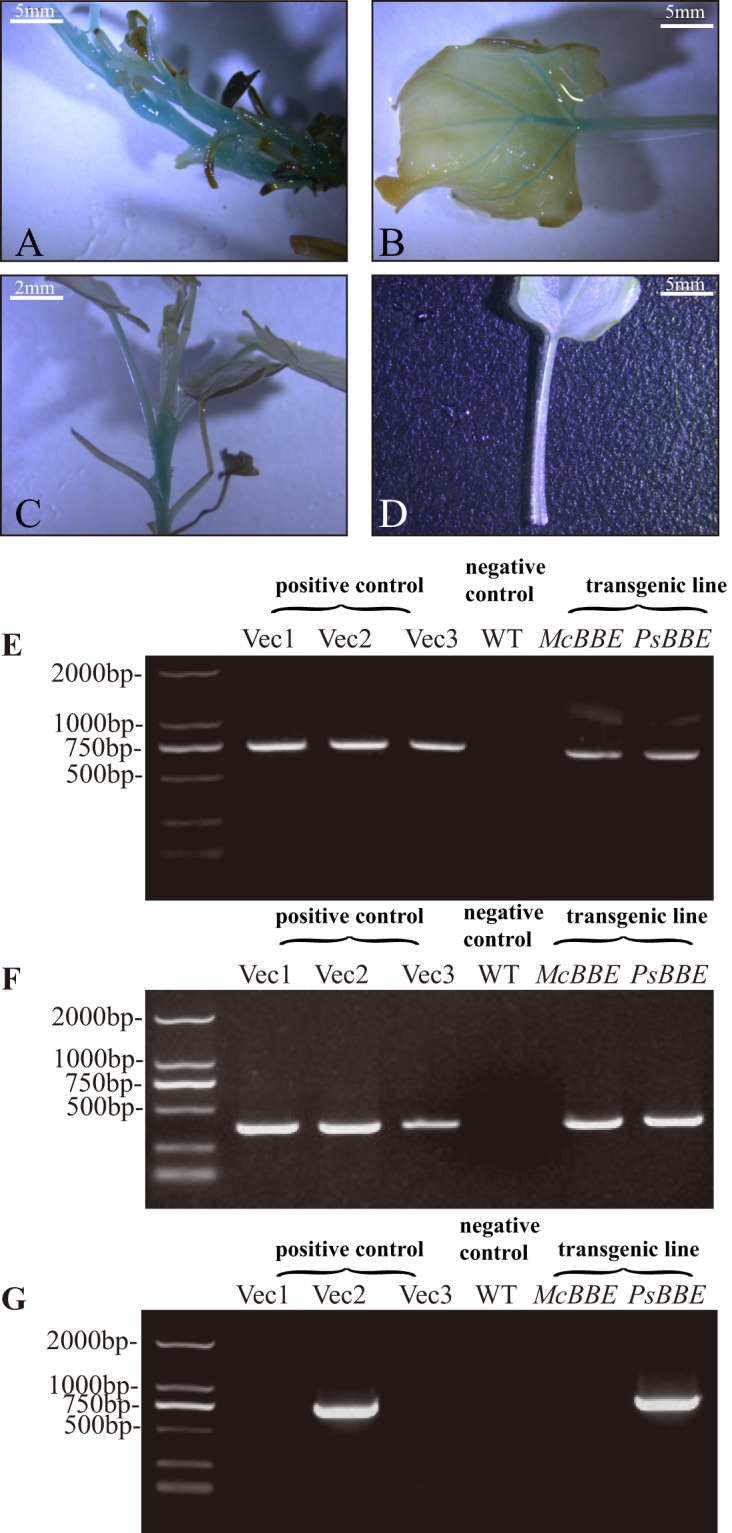
**(A**–**D)** GUS assay of leaf and stem section of transgenic and non-transgenic *M. cordata* plants. **(A,B)** Staining of GUS activity in overexpression McBBE-OX plants. **(C)** Staining of GUS activity in transgenic PsBBE-OX plant. **(D)** Staining of GUS activity in non-transgenic plant. **(E–G)** Amplify of *GUS, nptII, PsBBE* genes in transgenic and non-transgenic plants, marker 2 kb plus ladder and vector1 (pCMBIA-2301), vector2 (pCMBIA-2301 + *PsBBE*), vector3 (pCMBIA-2301 + *McBBE*) were used to positive control, WT negative control. **(E)** Amplify *GUS* gene (750 bp). **(F)** Amplify *nptII* gene (364 bp). **(G)** Amplify *PsBBE* gene (750 bp). Vec1-Vec3 means vector1 to vector3. WT means wild type.

We subjected leaf and stem explants to vacuum infiltration with *Agrobacterium tumefaciens* harbouring *McBBE-* or *PsBBE-* and *GUS* (β-glucuronidase)-overexpression vectors, respectively for 10 min, and then the explants were placed into co-cultivation medium (100 μM acetosyringone) for 3 days. Then, the explants were transferred to selection medium (75 mg/l kanamycin and 400 mg/l Timentin)^23^. To determine whether the transgenic lines were established successfully, GUS (β-glucuronidase) histochemical analysis was performed. The GUS histochemical results showed the presence of blue colour in the McBBE-OX and PsBBE-OX plants. In contrast, the wild-type lines did not show GUS activity (Fig. 2A–D). Polymerase chain reaction was performed using *GUS*, *nptII* (kanamycin resistance gene) and *PsBBE* primers for the transgenic lines, one wild type plant (negative control) and pCMBIA2301, pCMBIA2301 + *McBBE* and pCMBIA2301 + PsBBE plasmid vectors (positive control). The results showed that the reporter gene *GUS* and the *nptII* gene were detected in all three vectors and in all the transgenic lines (Fig. 2E–G). Only the 2301 + *PsBBE* vector and the PsBBE-OX line displayed *PsBBE*-specific fragments. No PCR amplification product was detected for the wild-type plants.

### Modulation of BIA biosynthesis by overexpression of McBBE and PsBBE in *Macleaya cordat*a and transcriptional activation of several genes encoding BIA biosynthetic enzymes

**Figure 3:**
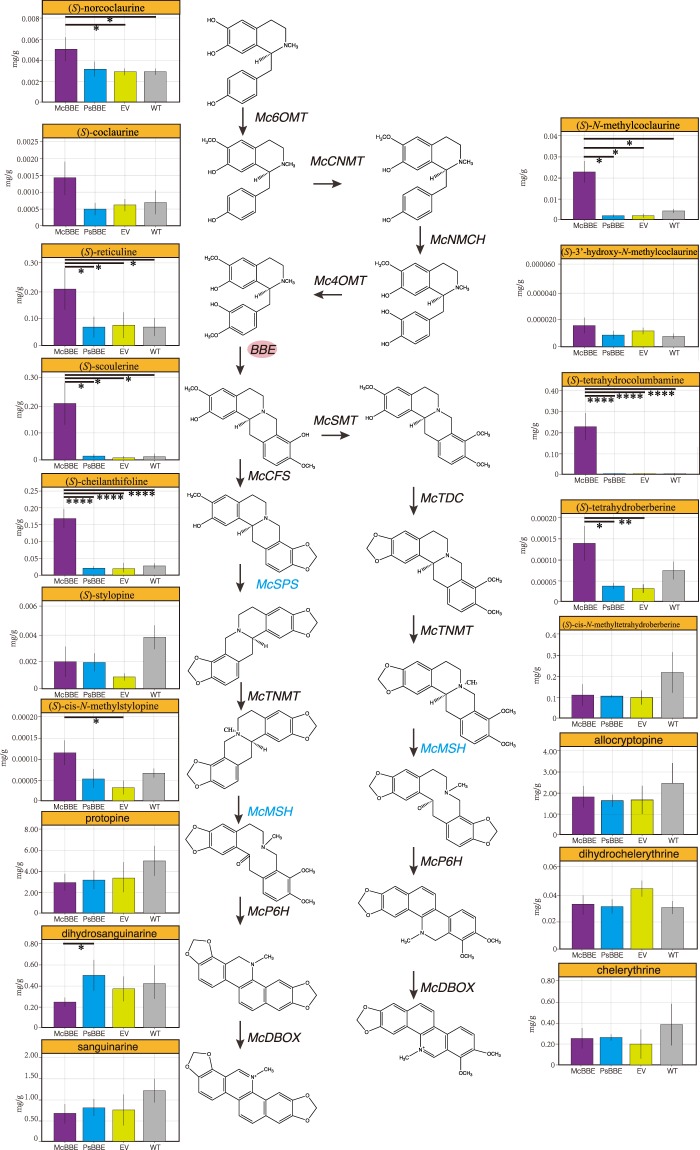
The analysis of alkaloid levels (from *(S)*-norcoclaurine to SAN and CHE) in transgenic (pcambia2301 + *McBBE*, pcambia2301 + *PsBBE*, pcmbia2301) and WT *M. cordata* plants were negative control. Data represent means ± SD (n = 3). Asterisks denote the significant changes (* means *P* < 0.05, ** means *P* < 0.01, *** means *P* < 0.005, **** means *P* < 0.001). *McMSH* and *McSPS* genes in this pathway still uncharacterized, we mark it in blue. EV means empty vector, WT means wild type.

We analysed the BIA content of 3-month-old plants using liquid chromatography with triple-quadrupole mass spectrometry (LC-QQQ MS) to detect whether BIA content was increased in the transgenic plants. Compared with those in the WT plants, in the McBBE-OX plants, the levels of (*S*)-norcoclaurine, (*S*)-*N*-cis-methylcoclaurine, (*S*)-reticuline, (*S*)-scoulerine, (*S*)-cheilanthifoline and (*S*)-tetrahydrocolumbamine were increased by 1.75-, 5.8-, 3-, 16-, 6- and 74-fold, respectively (P < 0.05). However, stylopine, PRO, dihydrosanguinarine (DHSAN), cis-*N*-methyltetrahydroberberine, allocryptopine (ALL), and chelerythrine (CHE) showed decreased concentrations upon the overexpression of *McBBE* (not significant, *P* > 0.05) (Fig. 3). However, there were no significant differences (*P* > 0.05) in the metabolic profiles among PsBBE-OX, EV and WT plants.

**Figure 4:**
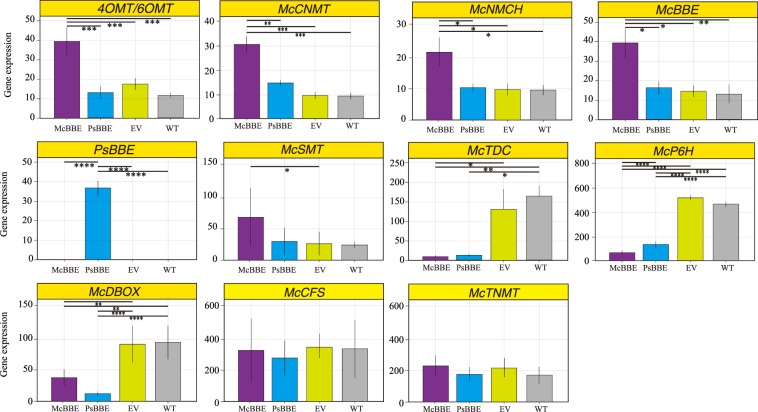
The analysis of gene expression levels in transgenic lines (pcambia2301 + *McBBE*, pcambia2301 + *PsBBE*, pcmbia2301) and WT lines. Data represent means ± SD (n = 3). Asterisks denote the significant changes (* means *P* < 0.05, ** means *P* < 0.01, *** means *P* < 0.005, **** means *P* < 0.001). EV means empty vector, WT means wild type.

Compared with that in the WT lines, the expression of the *Mc4OMT*, *Mc6OMT*, *McCNMT*, *McNMCH*, *McBBE* and *McSMT* genes in the McBBE-OX lines was significantly increased (Fig. 4). However, some other genes, *McTDC*, *McP6H*, and *McDBOX*, were significantly decreased. Since the *McBBE* gene was overexpressed in McBBE-OX lines, it is understandable that PsBBE-OX lines showed different results from McBBE-OX lines in the expression level of the *McBBE* gene. However, the results for PsBBE-OX plants were quite different from those for McBBE-OX lines and were more similar to those for the EV and WT lines, except regarding the expression of *McTDC, McP6H* and *McDBOX*.

## Discussion

One method to enhance target compounds in a secondary metabolic pathway is to overexpress the potential key enzyme at the genetic level. The BBE gene is responsible for a rate-limiting step in the biosynthetic pathway of BIA and affects the final production of BIA^12^. In the present study, the two BBE genes from *M. cordata* and *P. somniferum* were integrated into separate plant lines to analyse their impact on the BIA metabolic network. However, the levels of SAN in all transgenic plants (McBBE-OX, PsBBE-OX, and EV) were slightly lower than those in wild-type plants. This difference can be attributed to major changes in the levels of some intermediate metabolites; in particular, in the McBBE-OX line, some of the downstream metabolites, such as (*S*)-scoulerine, (*S*)-cheilanthifoline and (*S)*-tetrahydrocolumbamine, were significantly risen. Additionally, *McBBE* and *McSMT* gene expression was increased in the McBBE-OX line (*P* < 0.05), consistent with the metabolic profile. Then, we analysed the upstream metabolite profiles ((*S*)-norcoclaurine, (*S*)-coclaurine, (*S*)-N-cis-methylcoclaurine, and (*S)*-reticuline) and the expression levels of upstream genes (*Mc6OMT*, *McCNMT, McNMCH* and *Mc4OMT*) in the different transgenic lines (Figs 3 and 4). Compared with those in the PsBBE-OX, EV and WT lines, the expression levels of upstream genes (*Mc6OMT*, *McCNMT, McNMCH* and *Mc4OMT*) were significantly increased (*P* > 0.05) in the McBBE-OX lines. Correspondingly, the metabolite levels ((*S*)-norcoclaurine, (*S*)-coclaurine, (*S*)-N-cis-methylcoclaurine, and (*S)*-reticuline) in McBBE-OX lines were also higher than those in the other transgenic lines. A possible reason for the reduction in SAN content and the increase in reticuline content is that positive feedback derived from higher expression of the *McBBE* gene activates the expression of upstream genes. Furthermore, the reduction in the expression of downstream genes (*McTDC*, *McP6H*, and *McDBOX*) blocked the carbon flow and caused the upstream intermediate substances to accumulate slowly. A similar result was observed in 1-deoxy-D-xylulose 5-phosphate synthase (DXS)-overexpressing tissues in *Catharanthus roseus*^24^. These unintended consequences often appear when engineering single enzymes to increase flux. For example, overexpression of phytoene synthase in tomato increases lycopene content but downregulates the gibberellin pathway, ultimately resulting in dwarf plants. However, overexpression of BBE in *E. californica* hairy root culture increases total benzophenanthridines and slightly increases SAN content^12^, indicating that there are difference between species even when the same metabolic pathways are disturbed. On the other hand, the gene expression level of McTDC was significantly decreased in McBBE-OX lines, while the content of (*S*)-tetrahydroberberine in the McBBE group was the highest among all the groups (Fig. 3). Similarly, there were no differences between transgenic plants and wild-type plants in McCFS and McTNMT mRNA expression, but (*S*)-cheilanthifoline and (*S*)-tetrahydroberberine levels were significantly higher in McBBE-OX plants than in WT plants. The possible reason is that the accumulation of a large number of synthetic precursors in the McBBE-OX line resulted in higher levels of downstream products and caused a corresponding impact on metabolites. Through analysis of gene expression, we found that 9 genes were significantly influenced in transgenic plants, which affected the metabolite profiles (Figs 3 and 4). Another unexpected observation in this study was that transformation with PsBBE did not increase any BIAs. However, the overexpression of PsBBE in *Eschscholzia californica* significantly increases the levels of the end products^12^. Because the focus of this study was to use overexpression of McBBE to enhance BIA content, we will study this phenomenon in the PsBBE group in a future study.

Previous researchers have succeeded in achieving high production of BIAs by overexpressing the key enzyme in Papaveraceae plants^12–14^. However, it is difficult to cultivate many Papaveraceae plants; for example, the cultivation of poppy is regulated by the government. In contrast, *M. cordata* is free of addictive compounds and has a good prospect of cultivation^9^. The alkaloid profile in transgenic *M. cordata* was clearly made more diverse through overexpression of the key step gene and introduction of the exogenous gene. This article is the first to report the successful overexpression of key genes in *M. cordata*.

## Materials and Methods

### cDNA Synthesis and PsBBE synthetic

RNA from *M. cordata* root was isolated using the RNA prep pure Plant Kit (TIANGEN, CHINA) and reverse transcribed into cDNA using the PrimeScriptTM II 1st Strand cDNA Synthesis Kit (TAKARA, CHINA) according to the manufacturer’s instructions. The cDNA was used as a template for subsequent vector construction. The PsBBE (GenBankAF025430) synthetic from (Genscript, China).

### Plant Materials

The line originated from Fujian province (FJ1, available as seeds upon request; geographic coordinates: 27°46′23.408″N and 117°25′12.560″E) and cultivated at Hunan Agricultural University. Then further propagated via tissue culture to transgenic, metabolic analysis, and qRT–PCR, which would ensure the samples used in the following experiments have the same genetic background. All the tissue culture and the genetic transformation protocol for *M. cordata* was according to the previous paper^23^.

### Plant Expression Vector Construction

**Figure 5:**
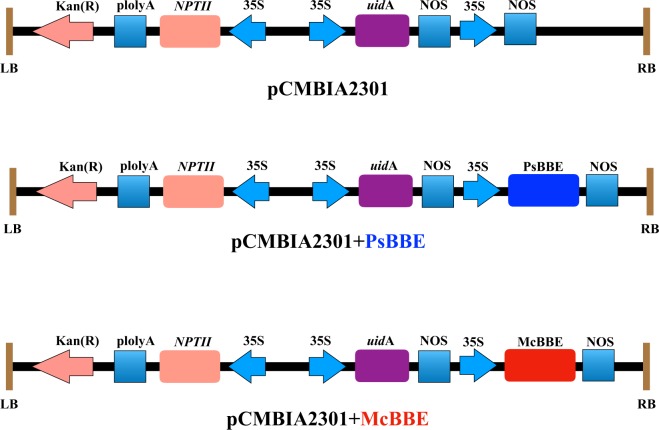
The plant expression vector pCAMBIA2301, pCAMBIA2301 + PsBBE, pCAMBIA2301 + McBBE used for *M. cordata* transformation. The T-DNA region contains the antibiotic selection marker, NPTII, driven by CaMV35 promoter and polyA signal. The uidA (GUS) reporter is under CaMV35S promoter and nopalinesynthase (Nos) terminator.

The plant expression vector pCAMBIA2301 (Fig. 5) was purchased from (Miaolingbio, China). McBBE and PsBBE was amplified using the primer listed in Table 1 by polymerase (NEB, Q5^®^ High-Fidelity DNA Polymerase, England). Then purified PCR products were subsequently cloned into pCAMBIA2301 using the In-Fusion Cloning Kit (Clontech, USA), and the pCAMBIA2301 was used as the empty vector control. These three vectors were introduced into *A. tumefaciens* strain GV3101, respectively for transformation in *M. cordata*.

**Table 1:** Nucleotide sequences of primers.

Primer Name	Oligonucleotide Sequences (5′-to 3′)
McBBE-plant-F	GTTGATAGCGGCCGCATGGATACAAAAATCAGAAAC
McBBE-plant-R	GATGTTTTGCCCGGGTTGTACAATTCCTTCTACATT
PsBBE-plant-F	GTTGATAGCGGCCGCATGATGTGCAGAAGCTTAACA
PsBBE-plant-R	GATGTTTTGCCCGGGCAATTCCTTCAACATGTAAA

### Genomic DNA Isolation and PCR Amplification

Genomic DNA was isolated from putative transgenic lines and wild type lines using TIANGEN DNeasy Plant Mini Kit (China, TIANGEN). The GUS (750 bp), npt II (364 bp) and PsBBE (750 bp) primers were used in PCR analysis to confirm the integration of T-DNA in transgenic lines formation (Table 2). Plasmid of pcambia2301 (positive control), pcambia2301-McBBE and pcambia2301-PsBBE and wild type (negative control). The PCR amplification program (China, TAKARA): denaturation at 94 °C for 5 min, followed by 30 cycles, 94 °C for 1 min, 58 °C for 30 second, and 72 °C for 30 second, final extension 72 °C for 5 min. The products were amplified products were analyzed by 1% (w/v) agarose gels prepared in 0.5XTBE (Tris/Borate/EDTA) buffer.

**Table 2:** Nucleotide sequences of primers.

Primer Name	Oligonucleotide Sequences (5′-to 3′)
npt II-F	TGCTCCTGCCGAGAAAGTAT
npt II-R	AATATCACGGGTAGCCAACG
PsBBE-F	GGAGCGATTCTTGACCGTGA
PsBBE-R	TCGACACGAACGGTTCCAAA
GUS-F	CTGGGTGGACGATATCACCG
GUS-R	GCGAAATATTCCCGTGCACC

### GUS Staining Analysis

We performed GUS histochemical staining in wild-type lines (WT) and putative transgenic lines. All tissues were infiltrated in phosphate buffer (pH 7.0, 0.1 M), EDTA (pH 8.0, 0.5 M), mannitol (0.3 M), X-Gluc (pH 7.0, 0.1 M) and 0.5% Triton X-100 for overnight at 37 °C. Then, Stained tissues were cleared in 70% ethanol.

### Metabolite Extraction and LC-QQQ MS Analysis

The WT and putative transgenic lines were collected after 3-month-old days of culture and ground into a fine powder using liquid nitrogen and then freeze-dried. Then, use ultrasonic extraction for 30 min at room temperature 1 mL of methanol, followed by ultrasonic extraction for 60 min at room temperature to isolate metabolites from 50 mg tissues. After filtration through a 0.22-mm membrane filter (Pall, USA), the solution was quantitative analyzed by LC/triple-quadrupole (QQQ) MS. An ultra-HPLC Agilent 1290 instrument coupled to a QQQ mass spectrometer (6460 A, Agilent) with a BEH C18 column (2.1 3 100 mm, 1.8 mm; Waters, Ireland) was used for the determination of 18 target alkaloids. The quantitative analyzed for metabolite according to our previous research^9^. The LC-QQQ MS data were processed using the Agilent Mass hunter Quantitative Analysis software (B.07.00). For absolute quantification analysis, the method was validated using the mixed standard solution, which was diluted with methanol to produce a at least 5 points and was used to evaluate absolute quantification of the target compound.

### Gene Expression Analysis by qPCR

Total RNA was isolated from putative transgenic line and wild-type of *M. cordata* using MiniBEST Plant RNA Extraction Kit (TaKaRa). The quality of RNA was checked by agarose gel electrophoresis and quantity was confirmed by Qubit 2.0. One micrograms of total RNA from each sample was reverse transcribed into cDNA using the PrimeScript RT reagent kit with gDNA eraser (TaKaRa, Dalian, China). The resulting cDNA products were diluted to 100 μL used as templates for subsequent experiments. PCR was performed on an ABI 7300 using FastStart Universal SYBR Green Master (ROX) according to the manufacturer’s instructions. The total volume of Quantitative real-time PCR assay was performed in a 20 μL (10 μL of PCR Mix, 0.5 μL of specific primers, 4 μL of cDNA and 5 μL of water). The qPCR cycling conditions were as follows: 95 °C for 15 min; 95 °C, 15 s; 55 °C 15 s; 72 °C 20 s, with 40 cycles. In this method, three replicates were performed in all cases. Relative gene expression was performed using the comparative 2^−ΔΔCT^method. We have been proved that only one gene involved in two methylation steps (*4OMT* and *6OMT*) in previous study. Therefore, the primers used in 4OMT and 6OMT are the same. All the primer sequence (Table 3) was checked via blast analysis and the *18S* gene as the internal reference.

**Table 3:** Nucleotide sequences of primers.

Primer Name	Oligonucleotide Sequences (5′ - to 3′ -)
*Mc6OMT/4OMT*-QP-F	CTGTTCCTAGTGCCCAAGCT
*Mc6OMT/4OMT*-QP-R	ACCACCAGTGTTCACCAACA
*McCNMT*-QP-F	CGATACGTTGGACGAGCAGA
*McCNMT*-QP-R	GGCCACACCCAAGATCAAGA
*McNMCH*-QP-F	ATCAACGCCTTGCTCATGGA
*McNMCH* -QP-R	GAGTTGGTGGGTGAAGTCGT
*McBBE*-QP-F	TTCACAGCCGGTTGGTGCCC
*McBBE*-QP-R	CCGCCTCGGATCGCCCAAAA
*McTDC*-QP-F	TGGTGGGACTGCCCTCCAAGT
*McTDC*-QP-R	TCAGGCATGTCGCGAGCTGAA
*McSMT*-QP-F	GCCGGGAAGGAGCCGAGGAT
*McSMT*-QP-R	TGATGCCGCGGATGTGTGGG
*McCFS*-QP-F	AATTTGGGTCGGTTCATGGC
*McCFS*-QP-R	GGCACCACCTTGAAGACCTT
*McTNMT*-QP-F	GCAGAGATAGCTTCGCACGA
*McTNMT-*QP-R	CCACATTCCACAGCTGAAGC
*McP6H*-QP-F	CATCAAGGACGTTCGAGCCT
*McP6H*-QP-R	CTCCTCACCACGCACAATCT
*McDBOX*-QP-F	ACTGTTGCCACGGTCGATAG
*McDBOX*-QP-R	TGGAGGAGCTTGTCAACACC
*PsBBE*-QP-F	GGAGCGATTCTTGACCGTGA
*PsBBE*-QP-R	TACTCCCCCAAGCACGGATA
*18S*-QP-F	CTTCGGGATCGGAGTAATGA
*18S*-QR-R	GCGGAGTCCTAGAAGCAACA

### Statistical Analysis

All the transgenic experiments were carried out in triplicate and each treatment contained 100 explants. All metabolic content and qPCR experiments were analyzed by one-way analysis of variance (ANOVA). The data were statistically analyzed using the GraphPad prism statistical software (version 7.0, USA). Differences between means were determined by analysis of variance with Tukey’s test on the level of significance declared at *P* < 0.05.
